# Impact of Magnetic Stimulation on Periodontal Ligament Stem Cells

**DOI:** 10.3390/ijms23010188

**Published:** 2021-12-24

**Authors:** Valentina Peluso, Laura Rinaldi, Teresa Russo, Olimpia Oliviero, Anna Di Vito, Corrado Garbi, Amerigo Giudice, Roberto De Santis, Antonio Gloria, Vincenzo D’Antò

**Affiliations:** 1 Department of Neurosciences, Reproductive and Odontostomatological Sciences, University of Naples Federico II, 80131 Naples, Italy; valentina.peluso2@unina.it (V.P.); olivier@unina.it (O.O.); garbi@unina.it (C.G.); 2 Department of Molecular Medicine and Medical Biotechnologies, University of Naples Federico II, 80131 Naples, Italy; laurarinaldi2000@yahoo.it; 3 Institute of Polymers, Composites and Biomaterials, National Research Council of Italy, V.le J.F. Kennedy 54. Mostra d’Oltremare Pad. 20, 80125 Naples, Italy; teresa.russo@cnr.it (T.R.); roberto.desantis@cnr.it (R.D.S.); antonio.gloria@cnr.it (A.G.); 4 Department of Experimental and Clinical Medicine, University Magna Graecia of Catanzaro, 88100 Catanzaro, Italy; divito@unicz.it; 5 Department of Health Sciences, School of Dentistry, Magna Graecia University of Catanzaro, 88100 Catanzaro, Italy; a.giudice@unicz.it

**Keywords:** magnetic stimulation design, stem cells, tissue engineering, osteogenesis, metabolomics, cellular respiration

## Abstract

The aim of this study was to evaluate the effect of a time-dependent magnetic field on the biological performance of periodontal ligament stem cells (PDLSCs). A Western blot analysis and Alamar Blue assay were performed to investigate the proliferative capacity of magnetically stimulated PDLSCs (PDLSCs MAG) through the study of the MAPK cascade (p-ERK1/2). The observation of ALP levels allowed the evaluation of the effect of the magnetic field on osteogenic differentiation. Metabolomics data, such as oxygen consumption rate (OCR), extracellular acidification rate (ECAR) and ATP production provided an overview of the PDLSCs MAG metabolic state. Moreover, the mitochondrial state was investigated through confocal laser scanning microscopy. Results showed a good viability for PDLSCs MAG. Magnetic stimulation can activate the ERK phosphorylation more than the FGF factor alone by promoting a better cell proliferation. Osteogenic differentiation was more effectively induced by magnetic stimulation. The metabolic panel indicated significant changes in the mitochondrial cellular respiration of PDLSCs MAG. The results suggested that periodontal ligament stem cells (PDLSCs) can respond to biophysical stimuli such as a time-dependent magnetic field, which is able to induce changes in cell proliferation and differentiation. Moreover, the magnetic stimulation also produced an effect on the cell metabolic profile. Therefore, the current study demonstrated that a time-dependent magnetic stimulation may improve the regenerative properties of PDLSCs.

## 1. Introduction

Periodontal ligament stem cells (PDLSCs) represent an innovative source of stem cells used for the development of regenerative therapies based on tissue engineering both in the dentistry clinical applications and in the reproduction or reconstruction of tissues. The periodontal ligament (PDL) is a system of connective fibers that surrounds the dental root and joins the root with the alveolar bone. PDL is a tissue characterized mainly of organized collagen fibers, called Sharpey’s fibers, inserting themselves into the bone and cementum. PDL performs several important physical, sensory and nutritional functions. The periodontal ligament is a tissue with a high turnover rate; the cellular elements include fibroblasts, endothelial cells, cementoblasts, osteoblasts, osteoclasts, tissue macrophages and stratified epithelial cells. 

PDLSCs are easily available, multipotent, with a high self-renewal capacity and consistent immunomodulatory effects. They are phenotypically and morphologically similar to mesenchymal stem cells (MSCs) [1,2,3]. In this way, they represent an ideal and alternative source of stem cells to those considered as the “gold standard” for the development of cell therapies and tissue engineering applications [4]. 

Human PDLSCs can be obtained from healthy donors by a minimally invasive procedure, and they are able to maintain the stemness features after long-term passages rather than the differentiation capacity [5]. 

Seo et al. in 2004 isolated, for the first time, PDLSCs from impacted human third molars and discovered their ability to differentiate into periodontal bones, cementum, alveolar ligaments, peripheral nerves and blood vessels [6,7,8]. The advantage is that these cells can differentiate into various cell lines as a consequence of the stimulatory agents used in their management [9].

They are used as a model for studying in vitro neurological diseases as well as for analyzing their differentiation potential and immunomodulatory properties. Moreover, human PDLSCs are easy to expand and manipulate in vitro, and they do not trigger a host immune response when transplanted [5].

It is well known that PDLSCs have an important role in maintaining periodontal homeostasis and are responsible for the regeneration and remodelling of periodontal tissues [8,10]. As reported in the literature, several biological mediators can influence cell phenotype and functions in the PDL and represent critical factors to understanding the homeostatic mechanisms regulating the regeneration of the PDL [11].

Various studies have clarified that mechanical forces, such as vibration, compression and tension, can significantly regulate the proliferation and differentiation of cultured PDLSCs, in vitro [12]. With regard to regenerative medicine, it is well known that in addition to mechanical stress/strain, other biophysical stimuli such as ultrasound and electromagnetic fields can improve tissue regeneration [13,14,15].

There is a long history of the clinical use of static and pulsed magnetic fields in the orthopedic field to increase bone fractures’ healing, to improve osseointegration of implants or to treat congenital pseudoarthrosis [16].

Furthermore, a magnetic field has been used in studies concerning drug delivery, hyperthermia for cancer therapy, magnetic stimulation of cell constructs and control of cell proliferation and differentiation [17,18]. 

Several studies in the literature have shown how the stimulation of a biocompatible material through a specific magnetic field may bring important advantages in terms of cell–material interaction [19,20]. 

Biochemical properties of stem cells and different cell populations may be influenced by an external static or pulsed magnetic field. Wang et al. in 2017 [21] showed that PDLSCs respond to a pulsed electromagnetic field (PEMF) stimulation improving the osteogenic differentiation but not the cell proliferation. It is also well recognized how the magnetic stimulation can lead to several effects according to different parameters such as waveform, frequency, intensity, duration and type of cell [22].

However, the mechanisms underlying the changes of the PDLSC properties induced by a magnetic field are still unclear. 

In this context, the effect of the combination design of a time-dependent magnetic field and poly(ε-caprolactone) (PCL)/Fe_3_O_4_ (80/20 *w*/*w*) nanocomposites on the behavior of mesenchymal stem cells was previously demonstrated [23]. 

Accordingly, the aim of the current research was to analyze the effect of a specific time-dependent magnetic field (6 h per day with 20 intervals of 18 min each) [23] on the behavior of PDLSCs, with a special focus on cell proliferation, differentiation and metabolic respiration.

## 2. Results

### 2.1. Effect of the Magnetic Field on Cell Proliferation

**Figure 1:**
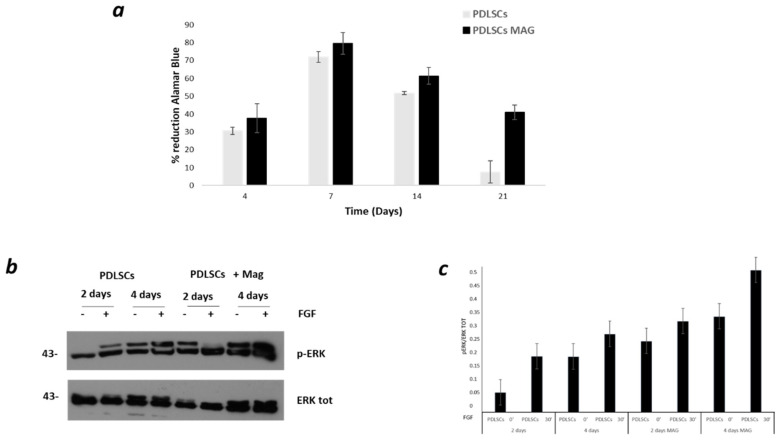
(**a**) Percentage of Alamar Blue reduction evaluated for PDLSCs and PDLSCs MAG at different time points. (**b**) Western blotting analyses performed on PDLSCs and PDLSCs MAG. The experimental groups were treated with fibroblast growth factor (FGF2) at indicated time points (0 and 30 min). (**c**) p-ERK normalized to the total amount of ERK for PDLSCs and PDLSCs MAG at 2 and 4 days.

A good cell viability was evident for both magnetically-stimulated PDLSCs (PDLSCs MAG) and unstimulated cells (PDLSCs) as the percentage of Alamar Blue reduction significantly increased over time up to 7 days showing a peak value at day 7. However, at 14 and 21 days after cell seeding, a significantly higher value of the percentage of Alamar Blue reduction was found in the case of PDLSCs MAG when compared to PDLSCs (*p* < 0.05), even if no statistically significant differences were observed at day 1 and 7 between the two groups (*p* > 0.05) (Figure 1a). 

A Western blot analysis showed the FGF-dependent ERK activation (Figure 1b,c). Furthermore, the magnetic field was able to stimulate a significant phosphorylation of ERK1/2, both in the presence and absence of FGF, in comparison to the control groups (PDLSCs) (Figure 1b,c). The ERK1/2 phosphorylation levels were dependent on the magnetic stimulation time, as evidenced by the significant increase of phospho-ERK1/2 levels between 2 and 4 days of exposure; nonetheless, the FGF treatment of PDLSCs MAG still contributed to a further activation of ERK1/2. 

### 2.2. Effect of the Magnetic Field on Cell Differentiation

**Figure 2:**
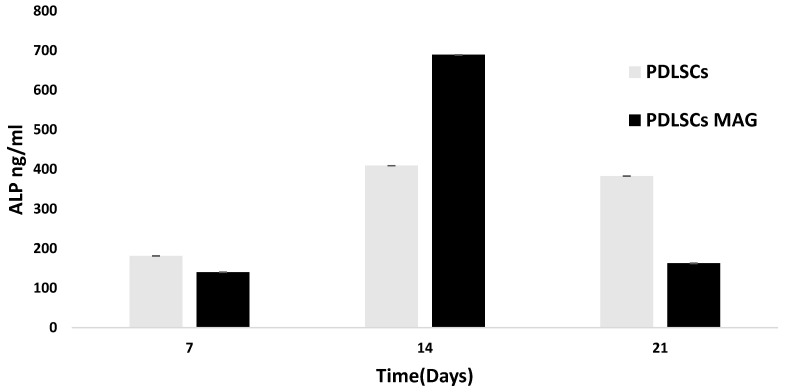
Alkaline phosphatase (ALP) activity for PDLSCs and PDLSCs MAG at 7,14 and 21 days after cell seeding.

The osteogenic differentiation was evaluated through the measurement of the ALP levels over time. In the case of PDLSCs MAG, the ALP showed a peak value at 14 days after the cell seeding (Figure 2). 

The decrease in the ALP activity from day 14 to day 21 could be due to a plateau value for the ALP level generally reached at day 21 and the onset of an advanced stage of osteogenesis indicated by later osteogenic markers. 

### 2.3. Effect of Magnetic Field on Cell Metabolism 

**Figure 3:**
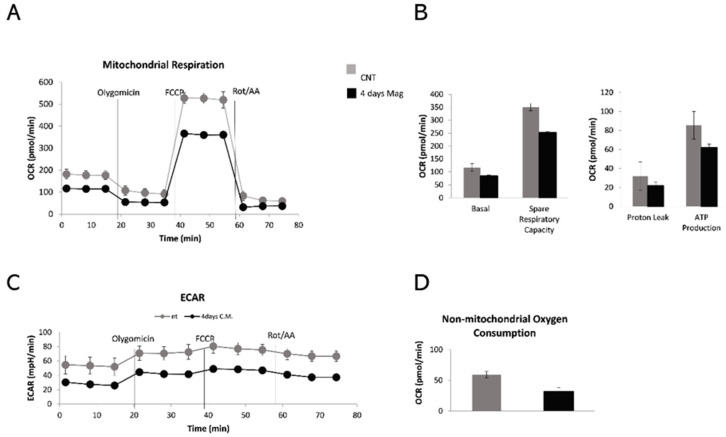
Respiration assays. (**A**) Mitochondrial respiration measured with sequential additions of oligomycin (1.5 μM), FCCP (1.0 μM), rotenone (3.0 μM). (**B**) Graphs represent the OCR rate; (**C**) ECAR rate; (**D**) non-mitochondrial oxygen consumption rate.

The oxygen consumption of cells was determined by using the Seahorse analyzer. Data are reported as oxygen consumption rate (OCR) and extracellular acidification rate (ECAR). The results showed a significant reduction in the baseline oxygen consumption of cells exposed to the magnetic field in comparison to the control cells. PDLSCs MAG showed a significant decrease in the ECAR level, lower non-mitochondrial oxygen consumption and, consequently, lower ATP production (Figure 3).

**Figure 4:**
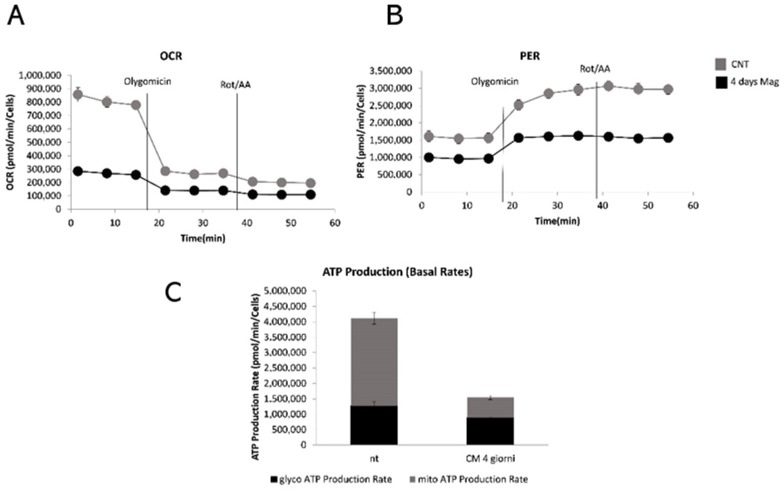
ATP rate assays. (**A**) Analysis of oxygen consumption. The rates of oxygen consumption (OCR) were measured with sequential additions of oligomycin (1.5 μM), rotenone (3.0 μM). (**B**) Proton efflux rate (PER). (**C**) ATP production rate.

Considering the decreased oxygen consumption rate, the glycolytic asset of PDLSCs MAG was monitored and compared to that of PDLSCs. Figure 4 reports the ATP production rates from mitochondrial respiration and glycolysis in real time.

Such an assay is able to detect the mitochondrial oxygen consumption rates (OCR) and proton efflux rate (PER), and, consequently, the mitoATP and glycoATP production rates. Thus, it allowed the quantifying of metabolic changes in response to a discontinuous magnetic field. Specifically, the data showed a slight decrease of glycolytic ATP production in PDLSCs MAG, when compared to PDLSCs, as well as a dramatic decrease of mitochondrial ATP levels in PDLSCs MAG.

The severe reduction of OCR followed by the decrease of mitochondrial ATP production after magnetic stimulation pushed the research towards a better understanding of the impact on the mitochondria physiological state. Accordingly, PDLSCs were exposed to the magnetic field for 4 days and the mitochondria state was checked through two different markers.

The MitoTracker Red is a red-fluorescent marker that stains the mitochondria of living cells, based on the mitochondrial membrane potential. The magnetic stimulation affected the membrane potential of mitochondria as shown by the lack of retention of the dye in PDLSCs MAG, resulting in a lower expression of red fluorescence.

To verify that mitochondria of PDCSCs exposed to the magnetic field were still healthy, the same experiment was performed with a different mitochondrial marker, HADHA, which is a fatty acid beta-oxidation enzyme.

**Figure 5:**
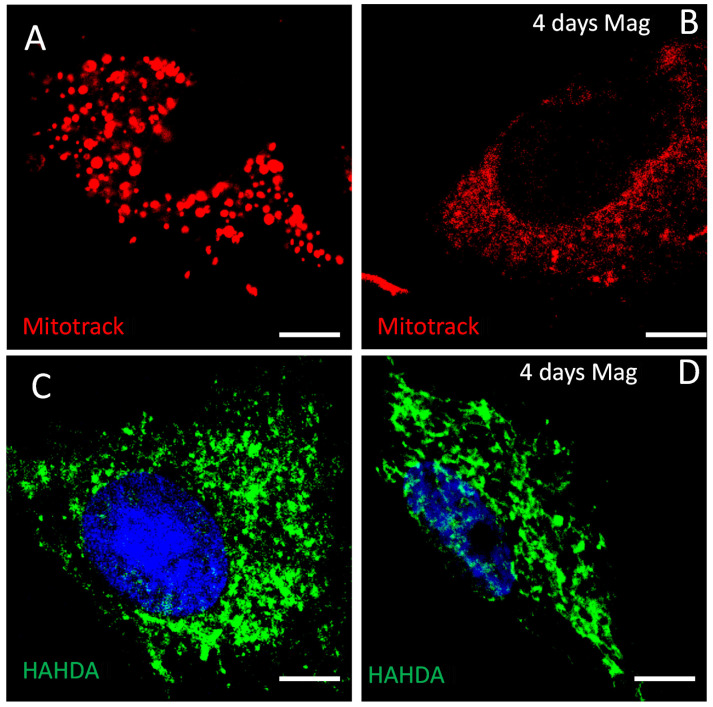
Confocal laser scanning microscopy analysis on PDLSCs MAG at day 4. (**A**,**B**) Images with MitoTracker Red fluorescence (red) of PDLSCs (upper left) and PDLSCs MAG (upper right). (**C**,**D**) PDLSCs immunostained with the antibody HAHDA (green) without (lower left) and with (lower right) magnetic stimulation. Scale bar: 100 µm.

The staining of HADHA in PDLSCs MAG showed that, despite the alteration of mitochondrial membrane potential, the magnetic stimulation did not significantly affect the mitochondrial morphology (Figure 5). 

## 3. Discussion

**Figure 6:**
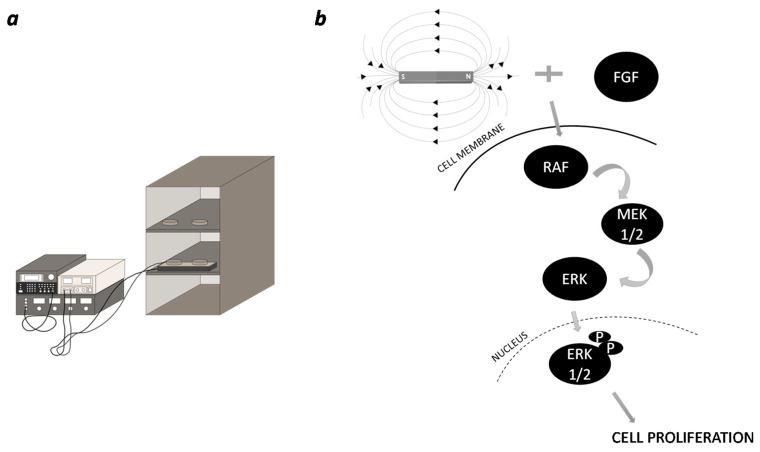
(**a**) Illustration of the magnetic device and of the incubator equipped with electromagnet. (**b**) Schematics illustrating the MAPK signalling pathway triggered by exposure to magnetic field.

In the current study, a novel approach to periodontal regeneration was provided. The synergic effect of a biophysical external stimulus (i.e., a time-dependent magnetic field) in combination with the osteogenic regeneration potential of PDLSCs, provided interesting results for future trends in the field of regenerative medicine and tissue engineering. Starting from the well-known MSC-like features of periodontal ligament stem cells (PDLSCs) [24,25], in the proposed research the changes due to the magnetic stimulation were analyzed in terms of proliferation, differentiation and cell metabolism. As reported in the literature, PDLSCs respond to PEMF stimulation [23] an electromagnetic field different from that used in the current study, even if the effects on the cellular behavior are controversial and still unclear [26,27], also depending on the employed parameters (intensity, frequency, exposure time) of the magnetic field. In this scenario, the main pathway involved in cell proliferation, apoptosis and differentiation was investigated: the mitogen-activated protein kinase (MAPK) cascade [28,29]. Therefore, to investigate the effect of a time-dependent magnetic field on periodontal ligament stem cells (PDLSCs MAG), the ERK1/2 phosphorylation on Thr202/Tyr204 was monitored in the course of the treatment with FGF, which is a well-known activator of the MAPK cascade (Figure 6).

As previously mentioned, the MAPK cascade is involved in several cell activities, also including cell differentiation. To investigate whether the increased activation of ERK, caused by magnetic stimulation, is also affecting PDLSCs’ osteogenic expression, the expression level of ALP was monitored. Alkaline phosphatase (ALP) is one of the most reliable markers for early osteogenic differentiation, as it is produced by osteogenic cells such as osteoblasts or by stem cells capable of osteogenic differentiation [30]. Moreover, considering the remarkable effects on cell growth and differentiation caused by the magnetic stimulation on PDLSCs, the metabolic state of cells exposed to MAG for 4 days was investigated.

The results showed a good cell vitality and a major proliferation activity of PDLSCs stimulated with the magnetic field when compared to the control cells. With or without FGF, ERK activation was observed in the presence of the magnetic field but the presence of FGF in conjunction with a magnetic exposure caused a greater activation of ERK1/2. These findings confirm that the application of a time-dependent magnetic field can improve the activation of the MAPK pathway. 

At the investigated time points, the significant increase in the ALP activity in PDLSCs MAG compared to cells without the magnetic stimulation suggested that the magnetic field induces the periodontal ligament cells to differentiate more effectively. Such results allowed the assumption that an external magnetic field induces the periodontal ligament cells to differentiate more effectively. Benefiting from the previous data, we wondered about if it was the metabolic state of PDLSCs stimulated by a discontinuous application of an external magnetic field. Our results showed that magnetic stimulation caused a decrease of oxygen consumption, a decrease of the extracellular acidification level and minor ATP production, which was mainly mitochondrial ATP. Nevertheless, the PDLSCs MAG mitochondria, observed through confocal laser scanning microscopy, did not appear morphologically damaged. Getting to know these metabolic data are important as energetic changes in cellular metabolism during glycolysis or OXPHOS oxidative reactions (states of hypoxia, glycolysis and redox) have an impact on stem cell differentiation, reprogramming [31,32], homeostasis and regeneration [33,34,35]. Hence, in the current investigation, a further insight was performed in the metabolic mechanism of PDLSCs in combination with a time-dependent magnetic stimulation. The obtained results would further stress the use of PDLSCs in regenerative medicine by improving their in vitro manipulation and promoting the migration into injured tissues [36]. 

However, potential limitations of the current study include: (i) no measurements of any interaction between PDLSCs and a suitably designed scaffold acting as a functional support for tissue engineering applications; (ii) a lack in the evaluation of the PDLSC behavior when using a synergistic combination of an externally applied magnetic field and a 3D nanocomposite scaffold with specific magnetic properties.

## 4. Materials and Methods

### 4.1. Cell Culture

Periodontal ligament stem cells (PDLSCs) were obtained from different patients aged 19–25 years (mean age 22.7 years). After the surgical extraction, impacted third molars were collected. PDL was collected by scraping the root surface from the middle third to the apical third, without involving apical papilla, and then minced. Small pieces of tissue were digested using 2 mg/mL collagenase type I solution (Gibco, Life Technologies, Italy) for 2 h at 37 °C. PDLSCs obtained from three PDLs of the same patient were seeded onto a plate with culture media and incubated at 37 °C in a humidified atmosphere of 5% CO_2_ to grow. The cells, characterized by flow cytometry, showed positivity for CD73, CD90 and CD105 while they were negative for CD14, CD45 and CD34 [24].

PDLSCs, at the fourth passage, were cultured in Dulbecco’s modified eagle medium high glucose (DMEM, Sigma Aldrich, Darmstadt, Germany) supplemented with 10% (*v*/*v*) fetal bovine serum (FBS, Gibco, Thermo Fisher Scientific, Waltham, MA, USA), 200 mM L-glutamine (Euroclone, Pero (MI), Italy) and antibiotics (penicillin G sodium 100 U/mL, streptomycin 100 mg/mL, Euroclone, Pero (MI), Italy). The cells, seeded on cell culture dishes, were incubated in a humidified atmosphere at 37 °C and 5% CO_2_. The cells were subcultured using trypsin/ethylene diamine tetraacetic (EDTA, Sigma Aldrich, Darmstadt, Germany). 

### 4.2. Magnetic Field Stimulation

Periodontal ligament cells were stimulated with a time-dependent magnetic field. One day after cell seeding, an external sinusoidal magnetic field with a frequency of 70 Hz and intensity of 30 mT was discontinuously applied for 6 h per day with 20 intervals of 18 min each [23]. An incubator equipped with an electromagnet was used; cell plates were placed over the electromagnet to expose cells to the magnetic field. To avoid mutual influence, the plates were placed at the right distance between them, about 10 mm apart. The non-magnetically stimulated plates used as the control were placed inside the same incubator.

### 4.3. Western Blot Analysis

PDLSCs, with a density of 1.2 × 10^5^ cells, were seeded in a 60 mm culture dish. Two and four days after magnetic stimulation (6 h per day for 20 intervals of 18 min each), the cells were serum-deprived overnight and left untreated, or treated with fibroblast growth factor (FGF2—10 ng/mL; Sigma Aldrich, Darmstadt, Germany) with treatment times of 30 min. The cells were collected with trypsin, centrifuged and pellets were obtained. The pellets were then lysed in a saline buffer containing 1 mM EDTA, 50 mM Tris-HCl (pH 7.5), 70 mM NaCl, 100 mM NaF and 1% Triton. The lysates were cleared by centrifugation at 15,000× *g* for 10 min and quantified by the Bradford method. An aliquot of lysed cells was resolved on sodium dodecyl sulfate polyacrylamide gel and transferred onto nitrocellulose membrane for 3 h. Filters were blocked for 1 h at room temperature in Tween-20 Phosphate buffer saline (TPBS; PBS- Sigma, 0.1% Tween 20, pH 7.4) containing 5% non-fat dry milk. Blots were then incubated O/N with the primary antibody. They were washed three times with a TTBS buffer and incubated for 1 h with a secondary antibody (peroxidase-coupled anti-rabbit) in TTBS-5% (*w*/*v*) non-fat dry milk. Reactive signals were detected by an ECL Western blotting analysis system. 

The autoradiography images were acquired and analyzed with Image-J software (NIH, Bethesda, MD, USA). The following primary antibodies were used: rabbit phospho ERK Thr202/Tyr204 (Cell Signaling) and rabbit ERK1/2 (Santa Cruz). Polyclonal antibodies were used at working dilutions of 1:1000.

### 4.4. Cell Proliferation Assay

Periodontal ligament stem cells (PDLSCs), at the fourth passage, were seeded in a 48 multi-well plate using a density of 1.0 × 10^4^ cells. To evaluate the proliferation and viability of magnetically stimulated PDLSCs (PDLSCs MAG) and unstimulated PDLSCs, the Alamar Blue assay (AbD Serotec Ltd., Kidlington, UK) was used. At 4, 7, 14 and 21 days after cell seeding, the cells were rinsed with PBS (Sigma Aldrich, Milan, Italy) and 200 µL of DMEM without phenol red (HyClone, Cramlington, UK) containing 10% (*v*/*v*) Alamar Blue was added to each sample. The samples were incubated for 4 h in a 5% CO_2_ diluted atmosphere at 37 °C. Subsequently, 100 microliters of the solution were removed from the wells and transferred to a 96-well plate. 

The assay was based on a redox reaction in the mitochondria of the cells, and the colored product was transported out of the cell. 

The optical density was measured through a spectrophotometer (Sunrise; Tecan, Mannedorf, Zurich, Switzerland) at wavelengths of 570 and 595 nm. The experiments were conducted three times in triplicate.

### 4.5. Osteogenic Differentiation Assay

Alkaline phosphatase (ALP) is an important marker for the early state of osteogenic differentiation in staminal cells. A specific enzymatic assay (SensoLyte pNPP Alkaline Phosphatase Assay Kit, AnaSpec Inc., Fremont, CA, USA) was used to evaluate the ALP activity. This assay was based on the substrate p-nitrophenyl phosphate (pNPP), a non-proteinaceous chromogenic substrate. PDLSCs were seeded into 48-well plates (density of 1.0 × 10^4^ cells) and subjected to magnetic exposure, whereas an unstimulated multi-well plate was used as a control. At 7, 14 and 21 days after the seeding, the cells were washed twice in PBS and lysed in 1 mL of lysis buffer. After collecting and centrifuging, the supernatant was used to calculate alkaline phosphatase (ALP). After a 30-min incubation with pNPP, the phosphatase was completely inhibited by NaOH and the para-Nitrophenylphosphate (pNPP) liberating inorganic phosphate and the conjugate base of para-nitrophenol (pNP). The resulting phenolate was yellow, with a maximal absorption at 405 nm. The ALP activity was directly proportional to the amount of pNPP liberated per unit time.

### 4.6. Metabolic Activity Assay

Periodontal ligament stem cells (PDLSCs), at the fourth passage, with a density of 1.2 × 10^5^ cells, were seeded in cell culture flasks. 

After 4 days of magnetic stimulation (6 h per day for 20 intervals of 18 min each) the cells, at the density of 6.0 × 10 ^4^ cells per well, were reseeded in triplicate into specific cell culture microplates (Agilent, USA) to be analyzed with two different metabolic kits: Seahorse XF Real-Time ATP Rate Assay Kit and Seahorse XF Cell Mito Stress Test Kit (Agilent, Santa Clara, CA, USA). 

Under the same conditions, cells not stimulated with the magnetic field were also analyzed. 

After 24 h, basal OCR was measured four times and plotted as a function of cells under the basal condition followed by the sequential addition of oligomycin (1 µg/mL), FCCP (1 µM) and rotenone (1 µM).

### 4.7. Confocal Laser Scanning Microscopy 

For the immunofluorescence study, unstimulated PDLSCs and PDLSCs stimulated with the magnetic field for 4 days were plated on poly-l-lysine coated (10 µg/mL) glass coverslips, fixed and immunostained with the primary antibody HAHDA. 

The immunoreactive signals were visualized by fluorescent-labeled secondary antibodies. The fluorescent signals were visualized using a Zeiss LSM 510 Meta argon/krypton laser scanning confocal microscope. 

Confocal images were acquired using a LSM 700 Zeiss confocal microscope (Carl Zeiss International, Göttingen, Germany). Images were captured using the ZEN software (Carl Zeiss International, Göttingen, Germany). 

### 4.8. Statistical Analysis

All experiments were independently repeated 3 times. The data were represented as means ± standard deviations. A one-way analysis of variance (ANOVA) followed by Bonferroni post hoc test was used for the statistical analysis. A value of *p* < 0.05 was defined as statistically significant.

## 5. Conclusions

Within the limitations of the current research, some conclusions were reached.

The time-dependent magnetic field was applied as an external biophysical stimulus determining relevant variations of PDLSC behavior, especially improving their proliferative and differentiation capacity. 

The presence of FGF in conjunction with an externally applied magnetic field caused a greater activation of ERK1/2. 

Furthermore, even though the magnetic stimulation can affect the mitochondrial membrane potential, the results also demonstrated that the mitochondrial morphology was not significantly altered.

The obtained findings would suggest the potential to promote the use of PDLSCs in regenerative medicine applications. Furthermore, the current study represents an initial step toward future research aiming to investigate the PDLSC–material interaction under magnetic stimulation.

## Data Availability

Not applicable.
